# Congenitally Corrected Transposition of the Great Arteries: Mid-term Outcomes of Different Surgical Strategies

**DOI:** 10.3389/fped.2021.791475

**Published:** 2022-02-03

**Authors:** Xiaomin He, Bozhong Shi, Zhiying Song, Yanjun Pan, Kai Luo, Qi Sun, Zhongqun Zhu, Zhiwei Xu, Jinghao Zheng, Zhifang Zhang

**Affiliations:** ^1^Department of Cardiothoracic Surgery, Shanghai Children's Medical Center Affiliated to Shanghai Jiao Tong University School of Medicine, Shanghai, China; ^2^Department of Cardiology, Shanghai Children's Medical Center Affiliated to Shanghai Jiao Tong University School of Medicine, Shanghai, China

**Keywords:** congenital heart disease, ccTGA, surgical strategies, anatomic correction, Fontan palliation, mid-term outcomes

## Abstract

**Background:**

Optimal management for congenitally corrected transposition of the great arteries (ccTGA) is controversial. We applied different surgical strategies based on individual variations in our single-centered practice over 10 years, aming to describe the mid-term results.

**Methods:**

From January 2008 to June 2021, 90 patients with ccTGA were reviewed and grouped by three different surgical strategies: 41 cases with biventricular correction as biventricular group, 11 cases with 1.5 ventricular correction as 1.5 ventricular group, and 38 cases with Fontan palliation as univentricular group. The mean age at primary surgery was 41.4 ± 22.7 months. Patients were followed for mortality, complications, reoperation, cardiac function, and valve status.

**Results:**

The median follow-up period was 5.1 years (range, 1.5–12.5 years). The overall 10-year survival and freedom from reoperation rate was 86.7 and 82.4%, respectively. There were 3 early deaths and 3 mid-term deaths in the biventricular group, while 2 early deaths and 1 mid-term deaths were reported in the univentricular group. Although 1.5 ventricular group presented no death and the fewest complications, we still found similar mortality (*p* = 0.340) and morbidity (*p* = 0.670) among the three groups. The bypass time, aortic-clamp time, and ICU stay length were the longest in the biventricular group, followed by the 1.5 ventricular group (*p* < 0.001). However, in mid-term follow-up, biventricular and 1.5 ventricular groups both showed excellent cardiac function and obvious improvement of tricuspid regurgitation (*p* = 0.008 and *p* = 0.051, respectively). Fontan palliation provided acceptable mid-term outcomes as well, despite a lower ejection fraction.

**Conclusion:**

Satisfactory mid-term outcomes could be achieved for highly selected ccTGA patients using the whole spectrum of surgical techniques. Moreover, 1.5 ventricular correction, as a new emerging technique in recent years, might hold great promise in future practice.

## Introduction

The surgical strategies for congenitally corrected transposition of the great arteries (ccTGA) have been evolving since 1860 when Rokitansky first described the disease as atrioventricular and ventricular-arterial discordance (1). Hitherto, anatomic correction has been accepted by more and more centers because accumulating evidence has shown its long-term benefit over physiological repair in suitable patients (2–4). The mid- to long-term results of biventricular anatomic correction are promising, since the morphologic left ventricle (mLV) and mitral valve (MV) are restored back into their systemic role, thereby significantly reducing the incidence of tricuspid regurgitation (TR) and failing right ventricle (RV) (5). However, the anatomic variability of ccTGA restrains the wide application of biventricular correction. In patients with unbalanced ventricles and cardiac malposition as well as other anatomic constraints, biventricular repair may not be possible. In these scenarios, Fontan palliation is still considered as an alternative. Despite the acceptable short- to mid-term results, for Fontan patients, multi-staged operation seems to be inevitable and the potential long-term quality of life is decreased (6).

In recent years, one-and-a-half (1.5) ventricular correction for partial anatomic correction also emerged as a promising choice (7). The strategy composes Glenn operation to unload the RV, hemi-Mustard at atrial level, and Rastelli/arterial switch operation (ASO) at ventricular level. Owing to its relative technical simplicity, 1.5 ventricular correction is especially suitable for complex patients with high risks to receive technical-demanding operations. Thus far, multiple benefits have been reported by a few experienced centers, including low mortality and complication rate in short-term follow-up (8, 9), yet the mid- to long-term results remain unknown.

Here, we present our single-center and over 10-year experience with different surgical strategies to treat ccTGA. By analyzing the follow-up results, we described the mid-term outcomes of different surgical strategies, aiming to reach a better and more suitable treatment for ccTGA.

## Patients and Methods

### Study Population

This retrospective study was carried out in Shanghai Children Medical Center and ethical issues were approved by Shanghai Children Medical Center Ethics Committee. From January 2008 to June 2021, a total of 90 consecutive ccTGA cases were collected. Patients who received physiologic repair were excluded. The mean age at primary surgery was 41.4 ± 22.7 months. ccTGA and associated lesions were diagnosed in all cases by echocardiography and enhanced computer tomography (CT) or magnetic resonance imaging (MRI) before surgery. Based on careful preoperational evaluations, 41 patients received biventricular correction as biventricular group, 11 patients received 1.5 ventricular correction as 1.5 ventricular group, and 38 patients received Fontan palliation as univentricular group. The baseline characteristics, morphology data, and associated anomalies are shown in Table 1.

**Table 1:** Baseline characteristics stratified by different surgical strategies.

	**Biventricular group** **(*n* = 41)**	**1.5 ventricular group** **(*n* = 11)**	**Univentricular group** **(*n* = 38)**	***P*-value**
**Baseline characteristics**				
Sex (male/female)	25/16	6/5	21/17	0.853
Age (month)	30.0 ± 21.4	48.6 ± 17.4	51.5 ± 19.1	**<0.001**
Weight (kg)	12.5 ± 4.4	13.5 ± 2.7	16.4 ± 3.9	**<0.001**
LVEF (%)	64.0 (62.0, 67.0)	61.0 (58.0, 66.0)	65.0 (62.0–68.0)	0.224
**Morphology data**				
Situs inversus	0	1 (9.0%)	2 (5.3%)	0.225
Dextrocardia	3 (7.3%)	3 (27.3%)	9 (23.7%)	0.090
Mesocardia	6 (14.6%)	3 (27.3%)	13 (34.2%)	0.126
**Associated cardiac**				
**anomalies**				
VSD	38 (92.7%)	11 (100%)	34 (89.5%)	0.057
LVOTO/PS	18 (43.9%)	8 (72.7%)	34 (89.5%)	0.512
Ebstein anomaly	3 (7.3%)	3 (27.3%)	3 (7.9%)	0.125
PH	10 (24.4%)	3 (27.3%)	5 (13.2%)	**0.006**
CoA/AA hypoplasia	1 (2.4%)	1 (9.1%)	1 (2.6%)	0.374
CAVC	0	0	4 (10.5%)	0.057
LSVC	0	2 (18.2%)	3 (7.9%)	0.225
Congenital heart block	2 (4.9%)	0	0	0.295
Straddling of TV	0	0	4 (10.5%)	0.057
Moderate TR	17 (41.5%)	6 (54.5%)	11 (28.9%)	0.245
Severe TR	5 (12.2%)	2 (18.2%)	4 (10.5%)	0.792

*LVEF, left ventricle ejection fraction; VSD, ventricular septal defect; LVOTO, left ventricular outflow tract obstruction; PS, pulmonary stenosis; PH, pulmonary hypertension; CoA/AA, coarctation of aorta/aortic arch; CAVC, complete atrioventricular canal; LSVC, left superior vena cava; TV, tricuspid valve; TR, tricuspid regurgitation. Statistical significance (p < 0.05) is shown in bold*.

### Surgical Indications and Techniques

**Figure 1:**
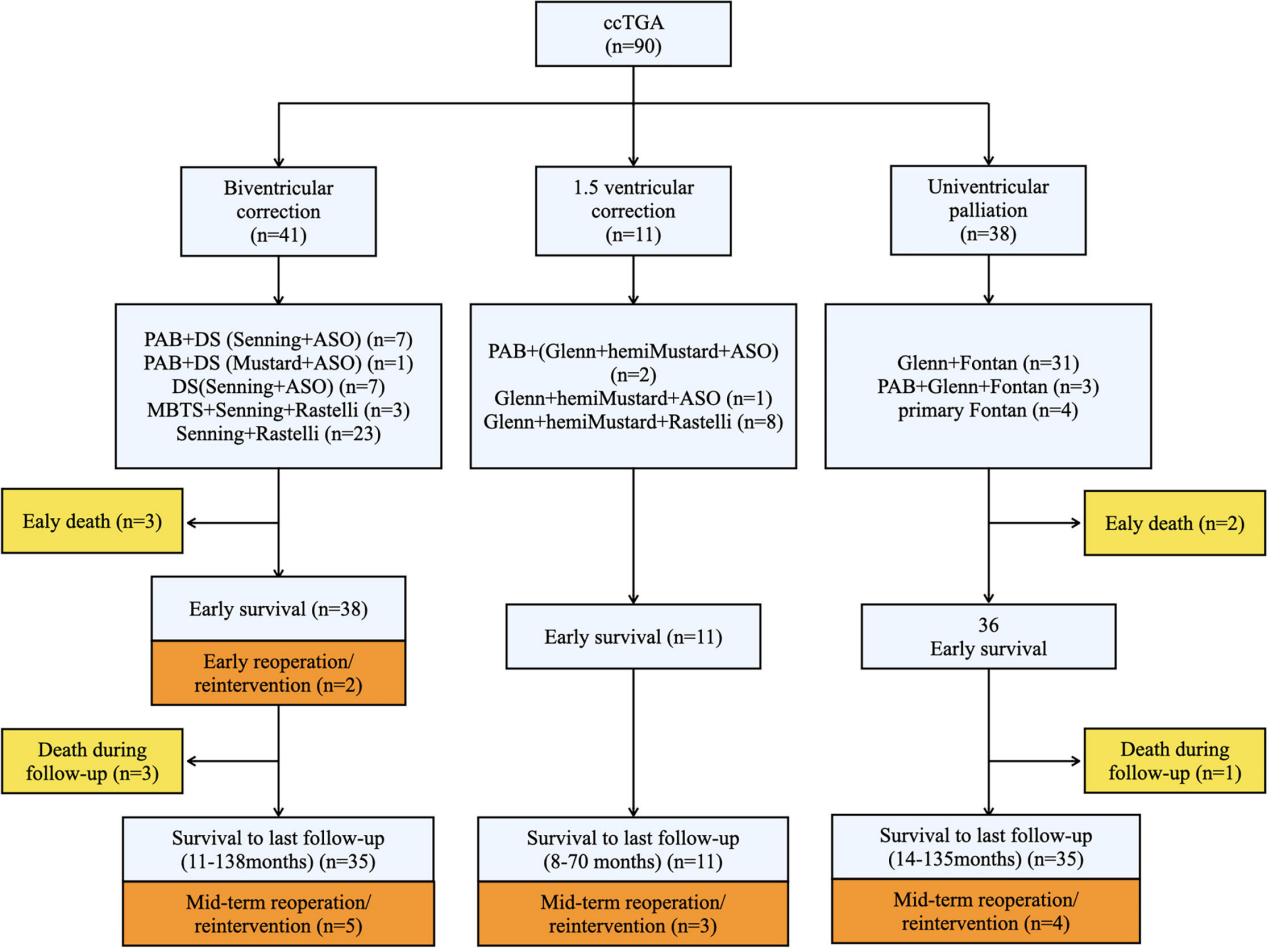
Schematic research route providing the overall illustration of our study. Three surgical strategies were adopted and the specific techniques were provided. Death and reoperation cases were shown in yellow and orange squares, respectively. The numbers of patients were indicatied in brackets of each squares. ccTGA, congenitally corrected transposition of the great arteries; PAB, Pulmonary artery band; DS, double switch; ASO, arterial switch operation; MBTS, modified Blalock-Taussig shunt.

A detailed illustration of patients with different surgical methods is shown in Figure 1.

### Biventricular Correction

The surgical strategies of biventricular correction were determined by key anatomic characteristics and functional status of the patients. In patients with well-developed pulmonary valve without obvious stenosis, double switch (DS) was performed. Arterial switch operation (ASO) was completed by standard coronary artery transplantation, arterial switch, and Lecompte procedure. At the atrial level, Mustard technique was used in early years, which was replaced by Senning technique in our later practice to avoid baffle obstruction and arrhythmia. Rastelli procedure was performed in patients with LVOTO or PS, briefly, and a right ventriculotomy was performed and a left ventricle to the aortic tunnel was established with a bovine pericardia baffle or a Dacron patch. The right ventricle–pulmonary artery (RV–PA) continuity was constructed by a bovine jugular vein conduit. In patients with severe PS and early cyanosis, modified Blalock-Taussig shunt (MBTS) was primarily performed to promote pulmonary development.

In patients without PS, a subgroup of selected patients with mild to moderate mLV hypoplasia and appropriate younger age, LV training was performed by pulmonary artery banding (PAB). The PAB was aimed to achieve an mLV/mRV pressure ratio of 0.66:0.75 assessed by piezometer in operation, as criteria established elsewhere (10). The decision whether to proceed with DS included mLV pressure maintained over 75% of systemic pressure for at least 2–3 months, no presence of ventricular dysfunction, and no moderate to severe mitral regurgitation. mLV mass index and mass/volume ratio were not used for measurement of inclusion.

### 1.5 Ventricular Correction

For patients with considerable small RV, cardiac malposition (dextrocardia and mesocardia), Ebstein's anomaly, or other forms of severe TR (assessed by echocardiography), 1.5 ventricular correction was preferred by our center as a partial anatomic correction. The 1.5 ventricular strategy we adopted here contained the hemi-Mustard, bidirectional Glenn, and Rastelli procedures. The hemi-Mustard operation was performed as standard. Briefly, the atrial septum was completely resected, the coronary sinus was unroofed, and an intra-atrial baffle was constructed by a pericardial patch to lead blood flow from the inferior vena cava and coronary sinus to the tricuspid valve. ASO or Rastelli procedure was then combined. Bidirectional Glenn was performed after de-clamping aorta to unload RV. PAB was performed in 2 patients as needed to control pulmonary hypertension.

### Fontan Palliation

After thorough evaluations such as reconstructed CT, cardiac catheterization, and even 3D printing in special cases, for patients with unfavorable anatomy who could not undergo anatomic correction, or the risk of anatomic correction was much higher than the benefit it may provided, we choose Fontan palliation as an alternative strategy. The indications of Fontan in our series included complex malformation of coronary arteries, insufficient morphology or dysfunction of the common atrioventricular valve, straddling of valves, CAVC with a severely hypoplastic ventricle, and DORV with remote VSD or with large VSD that were unable to establish intercardial tunnel. The univentricular repair was performed by staged operation or primary Fontan palliation in patients with late presentation.

### Functional Outcomes and Follow-Up

All patients received regular follow-up at 1, 3, 6, 9, and 12 months in the first year after discharge and once a year since. The median follow-up period was 5.1 years (range, 1.5–12.5 years). Mainly, a detailed Doppler echocardiography was used to evaluate the anatomy changes, valve regurgitation, and cardiac function during each assessment. Valve regurgitation (including tricuspid valve and common atrioventricular valve) was graded as none to mild, mild to moderate, moderate, moderate to severe, and severe. Additionally, ejection fraction was measured and New York Heart Association (NYHA) functional class were adopted to evaluate cardiac function.

### Statistical Analysis

IBM SPSS Statistics version 22.0 (IBM-SPSS Inc, Armonk, NY) was adopted to analyze our data. Briefly, the continuous variables that obey normal distribution were expressed by mean with SD, whereas skewed distributed variables were reported by median with range. ANOVA test with *post-hoc* correction was employed to compare continuous variables among three groups. Categorical variables were reported by frequency with percentage, and then the chi-square test or the Fisher's exact test was used to compare them. Kaplan-Meier curves and Cox regression were used to describe and compare survival and freedom from reoperation. *P* < 0.05 were considered statistically significant.

## Results

### Early Postoperative Outcomes

All patients in biventricular, 1.5 ventricular, and univentricular groups had satisfactory early outcomes. As shown in Table 2, we observed a significant difference among three groups in terms of operation time and in-hospital stay length (*p* < 0.001). The bypass and aortic cross-clamp time were significantly prolonged in biventricular group, indicating its complexity in surgical procedures, and was largely reduced univentricular group (*p* < 0.001). Moreover, the length of ICU stay and in-hospital stay were significantly reduced in the univentricular group as well (*p* < 0.001), but no difference was shown between biventricular and 1.5 ventricular group (*p* > 0.05).

**Table 2:** Early outcome stratified by different surgical strategies.

	**Biventricular group** **(*n* = 41)**	**1.5 ventricular group** **(*n* = 11)**	**Univentricular group** **(*n* = 38)**	***P*-value**
Bypass time (min)	245.1 ± 84.7	143.6 ± 34.2	114.2 ± 33.8	**<0.001**
Aorta cross–clamp time (min)	151.1 ± 36.8	79.6 ± 13.2	60.9 ± 23.9	**<0.001**
Mechanical ventilation (hours)	196.3 ± 81.9	152.6 ± 52.6	32.5 ± 50.7	**<0.001**
ICU stay length (day)	15.8 ± 6.7	12.6 ± 5.0	6.1 ± 3.3	**<0.001**
In–hospital stay length (day)	25.2 ± 9.5	23.7 ± 5.5	15.9 ± 7.2	**<0.001**
Survival to discharge	38 (92.7)	11 (100)	36 (94.7)	–
Early death	3 (7.3)	–	2 (5.6)	0.639
ECMO	2 (4.9)	–	–	0.295
Neurologic event	1 (2.4)	–	–	0.546
Acute renal failure	–	–	1 (2.6)	0.501
VAD implantation	1 (2.4)	–	1 (2.6)	0.866
Patients with ≥1				
Early complications	13 (31.7)	5 (45.5)	9 (23.7)	0.362
Bleeding	1 (2.4)	1 (9.1)	–	0.196
Pulmonary infection	1 (2.4)	–	1 (2.6)	0.546
Low cardiac output	3 (7.3)	1 (9.1)	3 (7.9)	0.981
Hypoxemia	3 (7.3)	2 (18.2)	2 (5.3)	0.367
Recurrent pleural effusion	5 (12.2)	3 (27.3)	4 (10.5)	0.340
RV dysfunction	–	1 (9.1)	–	**0.026**
Conduit obstruction	1 (2.4)	–	–	0.546
Diaphragmatic paralysis	1 (2.4)	–	–	0.546
Chylothorax	–	–	1 (2.6)	0.501
Need for early reintervention	1 (2.4)	–	–	0.866
Need for early reoperation	1 (2.4)	–	–	0.546

*ECMO, extracorporeal membrane oxygenation; VAD, ventricular assistance devices; RV, right ventricle. The definition of acute renal failure referred to urine output 0.3 mL/kg/h for 24 h (oliguria) or anuria for 12 h. Recurrent pleural effusion referred to refractory and persistent pleural effusion that unresponsive to diuretic treatments orally or intravenously, or the patients developed pleural effusion over 3 times in early postoperative period. The diagnosis of heart failure or low cardiac output was based on Society of Thoracic Surgeons (STS) database, as described elsewhere (11). Statistical significance (p < 0.05) is shown in bold*.

The early postoperative outcomes are shown in detail in Table 2. In the biventricular group, there were 13 (31.7%) cases with more than 1 early complications, including 3 cases with low cardiac output and 3 cases with hypoxemia due to recurrent pleural effusion. Moreover, two cases needed readmission and reoperation. One case with Senning + Rastelli operation developed obstructed intra-atrial baffle and received reoperation in 3 weeks after biventricular correction. Another case with Senning + ASO operation needed VAD implantation due to low cardiac output. Two patients needed ECMO due to low cardiac output but withdrew successfully, and they performed well during the follow-up period. In the 1.5 ventricular group, in total 5 cases had complications, including 2 cases with pleural effusion, one case with hypoxemia, one case with bleeding, and one case with right heart dysfunction. No reoperation was needed. There were 9 (23.7%) cases with important complications in univentricular group, including 4 cases with recurrent pleural effusion, 3 cases with low cardiac output, one case with chylothorax, one with pneumonia, and one case with acute renal failure due to hypoperfusion of the low cardia output. No early reoperation was needed as well.

Among 41 cases of biventricular group, three deaths (7.3%) occurred in the early postoperative period. One case died because of extremely low cardiac output after 3 days by Senning + ASO operation with additional IAA/VSD/PDA repairs. Despite a good general condition before surgery, the interrupted aorta was long and thin as indicated by preoperational enhanced CT, which might have contributed to his death. One death occurred on the second day after Senning + ASO operation with VSD closure because of acute renal failure and heart failure. The patient was also diagnosed as Ebstein anomaly with moderate TR, which did not improve after surgery and might be the reason for death. One case coexisted ventricular premature beat and I-II degree AVB before Senning + Rastelli procedure, who had recurrent malignant arrhythmia after surgery and died from ventricular fibrillation 8 days postoperatively. All 3 patients underwent thorough preoperational evaluation for the feasibility of biventricular conversion, including catherization and MRI, where the volume and pressure of the two ventricles were assessed to be adequate to withstand systemic circulation (all three patients had a LV/RV volume >30 ml/m^2^). There was no early mortality observed in the 1.5 ventricular group. In the univentricular group, two deaths (5.6%) occurred, including one case with multiorgan dysfunction and one case with supraventricular tachycardia. No out-of-hospital mortalities occurred in early outcomes.

### LV Training With PAB

In patients without PS, PAB was performed in 8 (53.3%) patients in the biventricular correction group, 2 (66.7%) patients in the 1.5 ventricular group. The mean age at PAB was 2.1 years old. The banding was placed to achieve an mLV/mRV pressure ratio of 0.66:0.75 in operation, and no cases developed an over-tightened banding before following operation. Two cases in the biventricular group recieved re-banding due to an unfavorable increase of mLV/mRV pressure. The banding was left in place before DS procedure for a mean duration of 14.6 ± 5.3 months. At the last evaluation before biventricular correction, the mean pressure gradient at the banding site was 57.3 ± 15.7 mmHg, and mLV/mRV pressure increased from pre-PAB 0.25 ± 0.11 mmHg to 0.72 ± 0.18 mmHg. All patients in the biventricular group maintained mLV pressure over 75% of systemic pressure for at least 2–3 months, and thus proceeded with DS procedure. One patient with restrictive VSD received PAB at 7.5 months age, since he had developed moderate TR at presentation and intended to receive biventricular correction. As a result, all 13 patients with PAB successfully received the following operation. No mortality was reported during the follow-up time span and all patients presented satisfactory cardiac function at the latest follow-up.

### Mid-term Follow-Up Outcomes

**Figure 2:**
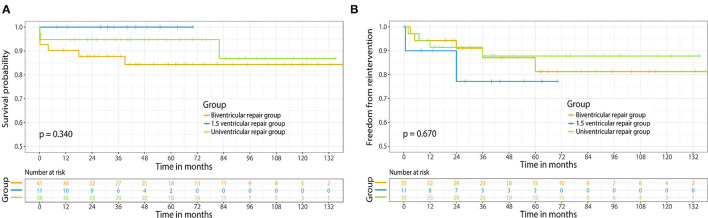
**(A)** Kaplan-Meier survival curve of different surgical strategies for ccTGA. **(B)** Freedom from reoperation of different surgical strategies for ccTGA. Numbers of patients at risk are indicated in the rectangle.

The median follow-up time of our study was 5.1 (range, 1.5–12.5) years. Survival was demonstrated in Figure 2A; in the biventricular group, one case succumbed to sudden death at home after 39 months. Two cases died because of heart failure. One of the patients was diagnosed as LV dysfunction and pulmonary infection 4 months after discharge, and despite the use of ECMO, the condition was not improved and the patient died from multi-organ failure and severe infection eventually. Another patient developed an obstructed intra-atrial baffle in 18 months follow-up and died from severe arrhythmia and LV dysfunction. The survival rates were 87.5, 83.9, and 83.9% at 1, 5, and 10 years, respectively. No mortality was observed during follow-up in the 1.5 ventricular group. The survival rates were 100%, 100% at 1 and 5 years, respectively. Only 1 mid-term death occurred in the univentricular group, who was diagnosed as low cardiac output and recurrent pleural effusion after 82 months follow-up, and subsequently died from multi-organ dysfunction. The survival rates were 94.7, 94.7, and 87.4% at 1, 5, and 10 years, respectively. The survival rates at 1, 5, and 10 years of the three groups were illustrated in Figure 2A; no significant difference was observed (*p* = 0.340).

An illustration of each case that needed reoperation is shown in Table 3. No mortality occurred in cases with reoperation. In the biventricular group, five cases with mid-term complications needed reoperation. The freedom from reoperation rates were 94.2, 79.7, and 79.7% at 1, 5, and 10 years, respectively. Two patients in the 1.5 ventricular group had mid-term complications that required reoperation. Due to limited follow-up span, the freedom from reoperation rates of 1.5 ventricular group were 90.0 and 76.2% at 1 and 5 years, respectively. In the univentricular group, 4 cases developed major complications, including 1 case underwent common atrioventricular valvuloplasty because of moderate to severe regurgitation. The freedom from reoperation rates were 91.3, 87.2, and 87.2% at 1, 5, and 10 years, respectively. As shown in Figure 2B, no significant difference was detected among three groups in aspect of the freedom from reoperation rates (*p* = 0.670).

**Table 3:** Short-to mid-term complications and reinterventions associated with each patient.

**Groups**	**Reoperation/reinterventions**	**Complications of each reoperation cases**	**Corresponding reinterventions/reoperations**	**Time since first correction (months)**
Biventricular group (*n* = 38)	5 (13.2)	III AVB	Pacemaker implantation	3
		III AVB	Pacemaker implantation	5
		LVOTO	LVOTO resection and enlargement	36
		LVOTO	LVOTO resection and enlargement	24
		RVOTO	Conduit replacement	60
1.5 ventricular group (*n* = 11)	3 (27.3)	RVOTO	Conduit replacement	24
		SVC thrombosis	Thrombectomy	0.75
		Diaphragmatic paralysis	Diaphragm plication	0.5
Univentricular group (*n* = 36)	4 (11.1)	Severe AVVR	AVV valvuloplasty	36
		III AVB	Pacemaker implantation	2
		SVC thrombosis	Thrombectomy	7
		Conduit obstruction	Conduit replacement	12

*AVB, atrioventricular block; LVOTO, left ventricular outflow tract obstruction; RVOTO, right ventricular outflow tract obstruction; SVC, superior vena cava; AVVR, atrioventricular valves regurgitation; AVV, atrioventricular valves*.

### Tricuspid Regurgitation and Cardiac Function

Since tricuspid regurgitation was reported to be one of the major concerns of ccTGA prognosis, we paid great attention to TR during follow-up (Table 4). The data excluded 4 patients with CAVC in univentricular group, the common atrioventricular valve insufficiency of whom remained at no improvement. All patients with more than moderate TR before surgery were improved. Within groups, all three groups presented satisfying TR improvement comparing pre- and postoperative data (*p* < 0.05). No moderate/severe TR was diagnosed in 1.5 ventricular group during follow-up, while only 3 cases occurred in the biventricular group, and 1 case in the univentricular group.

**Table 4:** Tricuspid regurgitation and cardiac function of the three groups comparing preoperative and last follow-up data.

	**Biventricular group (*****n*** **=** **41)**			**1.5 ventricular group (*****n*** **=** **11)**			**Univentricular group (*****n*** **=** **38)**		
	**Pre-operation**	**Latest follow-up**	***P*-value**	**Pre-operation**	**Latest follow-up**	***P*-value**	**Pre-operation**	**Latest follow-up**	***P*-value**
TR									
None to mild	19	26	**0.002**	3	10	**<0.001**	23	29	**<0.001**
Mild to moderate	3	6		–	1		4	2	
Moderate	14	3		6	–		4	1	
Moderate to severe	3	–		–	–		0	–	
Severe	2	–		2	–		3	–	
LVEF%	63.7 ± 4.32	65.9 ± 3.85	**0.008**	62.5 ± 5.07	65.7 ± 2.93	**0.051**	64.9 ± 3.77	64.1 ± 4.66	**0.509**

*TR, tricuspid regurgitation; LVEF%, left ventricle ejection fraction. Statistical significance (p < 0.05) is shown in bold*.

Mid-term follow-up data concerning cardiac function is also illustrated in Table 4. Obviously, patients in the biventricular group had significant improvement in LVEF% (*p* = 0.008) after surgery. The same trend was also observed in the 1.5 ventricular group though LVEF% elevation was not statistically obvious (*p* = 0.051), which probably resulted from the limited sample size. The postoperative LVEF% in the univentricular group, however, declined slightly with no statistical significance (*p* = 0.509). Moreover, only 2 patients were evaluated as NYHA grade III (1 case in biventricular and 1 case in univentricular group) in the follow-up period, while other patients all presented adequate heart function as NYHA grade I–II. There was no significant difference among three groups in NYHA cardiac function (*p* = 0.958).

## Discussion

Pursuing anatomical forms closest to normal physiology is not the only goal in treating ccTGA. On the contrary, best outcomes could be achieved by surgical strategies based on comprehensive factors (12, 13), such as individual anatomical variabilities and center experience. In our present study, all strategies including biventricular correction, 1.5 ventricular correction, and Fontan palliation for ccTGA showed favorable surgical treatments as well as mid-term results. The overall survival for ccTGA at 1, 5, and 10 years was 93.3, 90.5, and 86.7%, respectively, and the overall freedom from reoperation rate was 92.4, 82.4, and 82.4% respectively. No significant difference was observed. At the last follow-up, the vast majority of the patients presented excellent cardiac function and adequate valve performance.

Indeed, anatomical variabilities of ccTGA pose a great challenge to its surgical strategies. Physiologic repair was relatively common in early years and is still nonetheless a beneficial option in a subset of adult and adolescent patients with extreme complex anatomy or terminal RV dysfunction nowadays (12). Filippov et al. reported that the early mortality of DS procedure had no difference with that of physiologic repair (5). However, in mid- to long-term results, progressive TR and mRV failure were remarkable in physiologic repair, and thus the 10-year and 20-year survival were reported to be only 55 and 46% respectively (12). Since the late 1990s, more and more heart centers have accepted anatomic correction as an option with overwhelming superiority over physiologic repair in suitable patients (13, 14). In the current study, we also aimed to apply anatomic correction to whoever feasible. For ccTGA patients with VSD and/or PS, we preferred to perform biventricular correction if possible. Corresponding results were presented in our series; however, we also found a considerable morbidity of early complications of DS procedure, which was due mainly to intra-atrial baffle obstruction and cardiac block. Helbing and Karl reported a 5–7% rate of intra-atrial baffle obstruction after Senning or Mustard procedure (15, 16), corresponding to 4.8% baffle obstruction rate of biventricular correction group in our study. A modified Senning procedure was recently proposed that could possibly reduce the obstruction rate (17), yet still needs longer follow-up to be verified. To reduce the need of valved conduit replacement and pacemaker implantation, some centers proposed that patients without obvious symptoms could wait until their preschool age to receive DS operation (18). Lastly, it is noteworthy that we found prolonged CPB time, aortic cross-clamp time, and mechanical ventilation time in the biventricular group compared to the 1.5 and univentricular groups. This is likely due to the technical complexity associated with biventricular repair. The technical complexity also, to a very large extent, contributed to the three early deaths in the biventricular group.

For patients with PS and severe cyanosis, we preferred a first staged systemic-pulmonary shunt before DS procedure. For patients without PS, the timing of operation depends partly on the size of VSD. Operation should be carried out as soon as possible in patients with restrictive VSD or without VSD. Under circumstances with untrained LV, these patients develop rapid LV degradation and hypoplasia as the low pulmonary afterloads hardly maintain enough pressure for LV development. Many of these patients developed severe TR and RV dysfunction accumulated with time, and have lost the opportunity of biventricular correction. In our study, one case with restrictive VSD received PAB soon after he presented at clinic, in consideration of the moderate TR he had developed. Whenever a significant LV hypoplasia exists, PAB should be performed to train LV before DS. Myers et al. suggested that the best age for LV training should be <2 years old. Otherwise, patients elder than 12–15 years old might lose such opportunity and increase the risk of neo-aortic valve regurgitation (19). Preventive PAB at newborn age was also proposed by some centers, in order to prevent LV degeneration and mRV failure (20). In our cases, the mean age for LV training was 2.1 years old. Some centers suggested a moderate PAB with 50–60% systemic pressure to increase after-load and a creation of ASD fenestration to increase pre-load, for training LV function gradually to protect the valves (21, 22). In our practice, all patients in biventricular group maintained mLV pressure over 75% of systemic pressure for at least 2–3 months. All patients with PAB showed satisfactory mid-term outcomes in the follow-up period.

However, DS operation is not suitable for all ccTGA patients. In patients with cardiac malposition, severe right ventricular hypoplasia, Fontan operation was traditionally considered as an alternative option. Satisfactory early survival of Fontan operation in treating ccTGA was well-demonstrated, but due to Fontan related complications in long term, the life quality of univentricular patients was not ideal (6). For this reason, Fontan palliation is not supported by the prevailing opinions unless there is no alternative.

In our series, the Fontan operation was recommended for patients with limited left ventricular outflow tract, insufficient morphology, or dysfunction of the common atrioventricular valve, and those who could not (or with high risks to) receive biventricular correction. Because Fontan palliation is a technically simpler operation, the complications and reoperation of Fontan palliation group were fewer than those of anatomic correction groups. Though the overall TR improvement in univentricular group was observed and significant in statistics, we partly believed this improvement could mainly be attributed to PAB instead of Fontan itself. Not surprisingly, LVEF% assessment showed that Fontan pathway provided the worst cardiac output, yet in an acceptable range. There were no differences in outcomes between staged and primary Fontan in our study. These results might indicate that, in mid-term perspective, Fontan has never lost its competitiveness in treating specific groups of patients with ccTGA, albeit some inherent problems that may affect life quality in long term. Although anatomic correction was primarily proposed to achieve better long-term outcomes by the mainstream views, Fontan palliation could still be a rational strategy in chosen patients and centers with less experience.

For a portion of ccTGA patients, biventricular correction might be performed with extreme risks whereas the long-term outcomes were poor with univentricular repair. Hence, Baylor College of Medicine first offered a modified DS procedure in 2004 (23), using hemi-Mustard and Rastelli operation with bidirectional-Glenn procedure to achieve 1.5 ventricular correction. With broad indications, 1.5 ventricular correction particularly fits patients with unstable Rastelli, right ventricular hypoplasia or dysfunction, or moderate to severe TR. Hemi-Mustard avoids the suture across the sinus node, which might be beneficial to preserve sinus node comparing to traditional Senning or Mustard (8). In addition, the bidirectional-Glenn relieves right ventricular load and reduces blood flow from left ventricle to pulmonary artery (24). Besides, the simplicity of the technique itself largely reduces perfusion and ischemic time, even in situations of cardiac malposition. In our series, there was no mortality in the 1.5 ventricular group. All patients had excellent LVEF% and NYHA cardiac function at the latest follow-up. All patients presented TR less than moderate after operation, even in 2 severe cases preoperationally, significantly superior to univentricular repair. Moreover, the CPB time of 1.5 ventricular group showed no statistical difference with the univentricular group and was significantly lower than the biventricular group. Thus, taking comprehensive factors above into consideration, 1.5 ventricular correction seems to hold great promise in mid-term outcomes in treating ccTGA, yet the long-term outcomes and life qualities remain to be investigated. Even so, to some degree, 1.5 ventricular correction broadens the indications for anatomic correction. Patients with unfavorable anatomy who were deemed to receive single ventricular fate in the past now have a chance to receive partial anatomic correction.

### Limitations

Only recently, 1.5 ventricular correction was carried out in our center, and thus the number of patients in 1.5 ventricular group as well as follow-up period was limited. To further legitimize the efficacy of 1.5 ventricular correction in ccTGA, a larger sample size and a longer follow-up span are needed. To further illustrate outcomes of Fontan palliation, functional outcomes beyond the NYHA classification were needed, which might provide information related to exercise tolerance, complications related to Fontan circulation, and life qualities. In addition, it would be more solid and persuasive to have catherization and MRI data in follow-up examinations, which described cardiac-output, mass-volume ratio, and LV:RV pressure in detail. Due to unbalanced distribution of different groups and selection bias, the statistical analysis was not strictly powered, and thus we tried not to compare the three groups but only focus on the description of the results.

## Conclusion

The surgical strategies for ccTGA should be tailored according to individual situations. In our practice over the recent 10 years, although we could not reach biventricular correction for all patients, the mid-term outcomes were also satisfactory in selected patients with 1.5 ventricular correction and Fontan palliation. Notably, in concern of the non-negligible drawbacks of the biventricular correction and Fontan palliation, 1.5 ventricular correction is nonetheless an alterative with excellent mid-term results, yet its long-term benefits remain to be determined.

## Data Availability Statement

The original contributions presented in the study are included in the article/supplementary material, further inquiries can be directed to the corresponding author/s.

## Ethics Statement

The studies involving human participants were reviewed and approved by Shanghai Children Medical Center Ethics Committee. Written informed consent to participate in this study was provided by the participants' legal guardian/next of kin. Written informed consent was obtained from the minor(s)' legal guardian/next of kin for the publication of any potentially identifiable images or data included in this article.

## Author Contributions

XH and BS: conceptualization and writing—original draft. ZS: data curation and statistical analysis. YP and KL: formal analysis. QS: resources. ZZhu, ZX, and JZ: surgical decision and operation. ZZha: conception and patients' follow-up. All authors contributed to the article and approved the submitted version.

## Funding

This study was supported by the Clinical Research Plan of SHDC (SHDC2020CR4093), General Project of Clinical Research in health industry of Shanghai Health Committee (202040337), Shanghai Municipal Science and Technology Commission (19411963700), and the National Key R&D Program of China (2017YFC1308100).

## Conflict of Interest

The authors declare that the research was conducted in the absence of any commercial or financial relationships that could be construed as a potential conflict of interest.

## Publisher's Note

All claims expressed in this article are solely those of the authors and do not necessarily represent those of their affiliated organizations, or those of the publisher, the editors and the reviewers. Any product that may be evaluated in this article, or claim that may be made by its manufacturer, is not guaranteed or endorsed by the publisher.

## Abbreviations

ccTGA: congenitally corrected transposition of the great arteries
mLV: morphologic left ventricle
MV: mitral valve
RV: right ventricle
TR: tricuspid regurgitation
ASO: arterial switch operation
TV: tricuspid valve
PAB: Pulmonary artery banding
MBTS: modified Blalock-Taussig shunt
DS: double switch
LVEF: left ventricle ejection fraction
VSD: ventricular septal defect
LVOTO: left ventricular outflow tract obstruction
PS: pulmonary stenosis
PH: pulmonary hypertension
CoA/AA: coarctation of aorta/aortic arch
CAVC: complete atrioventricular canal
LSVC: left superior vena cava
ECMO: extracorporeal membrane oxygenation
VAD: ventricular assistance devices
AVB: atrioventricular block
LVOTO: left ventricular outflow tract obstruction
RVOTO: right ventricular outflow tract obstruction
SVC: superior vena cava
AVVR: atrioventricular valves regurgitation
AVV: atrioventricular valves.
